# Clinical and economic outcomes of a pharmacogenomics-enriched comprehensive medication management program in a self-insured employee population

**DOI:** 10.1038/s41397-024-00350-1

**Published:** 2024-10-02

**Authors:** Maren S. Fragala, Murray Keogh, Steven E. Goldberg, Raymond A. Lorenz, Jeffrey A. Shaman

**Affiliations:** 1 Quest Diagnostics, Secaucus, NJ USA; 2 Coriell Life Sciences, Philadelphia, PA USA

**Keywords:** Health services, Health care

## Abstract

Clinical and economic outcomes from a pharmacogenomics-enriched comprehensive medication management program were evaluated over 26 months in a self-insured U.S. employee population (n = 452 participants; n = 1500 controls) using propensity matched pre-post design with adjusted negative binomial and linear regression models. After adjusting for baseline covariates, program participation was associated with 39% fewer inpatient (p = 0.05) and 39% fewer emergency department (p = 0.002) visits, and with 21% more outpatient visits (p < 0.001) in the follow-up period compared to the control group. Results show pharmacogenomics-enriched comprehensive medication management can favorably impact healthcare utilization in a self-insured employer population by reducing emergency department and inpatient visits and can offer the potential for cost savings. Self-insured employers may consider implementing pharmacogenomics-enriched comprehensive medication management to improve the healthcare of their employees.

## Introduction

A compelling case has been made that pharmacogenomic-enriched comprehensive medication management (PGx + CMM) [1] is ready to become a clinical standard of care and has the potential to provide all stakeholders with an approach to addressing medication safety, poor health, and rising healthcare costs. [2] The scalable, broad utilization of genetic testing in personalized medicine requires five factors working together—clinical utility, laboratory technology, user acceptance, implementation models, and economic value—to achieve value for patients, providers, and payors, and to avoid disruption of existing clinical workflows. [2] Specifically, the tipping point has been reached in favor of population-level, large-scale pharmacogenomic testing. This study adds information about clinical and economic impacts in a self-insured employee population.

Pharmacogenomics, as a tool in the clinical practice of medicine, helps healthcare providers optimize drug selection and dosing, avoid adverse events, and identify responders and non-responders to medications [3]. The US Food and Drug Administration (FDA) details a list of over 500 drug-gene (biomarker) pairs with pharmacogenomic information included in drug labels for a variety of therapeutic applications including mental health, cardiology, pain management, diabetes, gastroenterology, neurology, chemotherapy, and infectious diseases [4]. The genes appearing in drug labels and professional guidelines—and recommended for laboratory testing—are well-described components of metabolic enzyme pathways, cell membrane transport mechanisms, and chemical receptors and their downstream cell signaling pathways [5–8].

With the delivery of CMM, the promise of personalized medicine is realized by identifying the most effective and safe therapeutic regimen through the assessment of genetic and other medication therapy risk factors. These factors include concurrent medications and medical conditions, age, diet, smoking status, and adherence [9]. A recent real-world implementation of a PGx + CMM program showed economic and clinical outcome improvements in a Medicare-eligible population [1]. We previously demonstrated the feasibility of PGx + CMM in a self-insured employer setting and described existing population risks and opportunity to improve medication management. The research demonstrated that 86% of employees who completed the program received actionable recommendations, averaging 5 recommendations per person [10].

The present study compared economic and clinical outcomes between participants in a PGx + CMM program with matched controls. The primary hypothesis of the study was that there is an association between the PGx + CMM intervention and changes in healthcare resource utilization (HRU) yielding reduction in medical costs as measured by claims data at 13 months post-program inception.

## Methods

### Study design and setting

The impact of a self-insured employer-sponsored PGx + CMM program on HRU and medical costs was evaluated using a retrospective cohort pre-post design. Medical and pharmacy claims data from February 2020 to February 2021 were used to assess risk in individuals and invite those identified as high-risk, based on potential for drug-drug interactions, anticholinergic burden, contraindications, and medications impacted by genetics. Following enrollment which began in March 2021, Quest Diagnostics provided genotyping services and the results were transferred to Coriell Life Sciences for clinical annotation and interpretation. The retrospective data analysis was conducted using medical and pharmacy claims data from February 2020 to March 2022 for consented participants. All components of the program and study were performed under an approved IRB protocol (Biomedical Research Alliance of New York Institutional Review Board; BRANY).

### Study population and participant engagement

Eligible employees ≥18 years old were invited to participate in the program. Employees were deemed eligible if they were enrolled in the employer’s sponsored medical plan, and were ranked as high-risk, based on a weighted aggregation of the calculated risks for potential drug-drug interactions, anticholinergic burden, contraindications, and medications impacted by genetics from pharmacy and medical claims records in the 12 months immediately prior to the start of the program. Medications impacted by genetics were derived from FDA package labeling, the FDA Table of Pharmacogenetic Associations, Clinical Pharmacogenetic Implementation Consortium (CPIC) guidelines, and literature reviews. This includes medications impacted by genetic polymorphisms or variants known to affect drug metabolism enzymes, drug transporters, or drug targets and have met specific inclusion criteria. Enrollment consisted of a web-based survey or phone call to collect contact, medication, diet, and lifestyle (e.g., smoking) information. Additional education and outreach were also part of the ongoing recruitment process.

### Genetic testing

The enrollee self-collected saliva sample was shipped to and analyzed by a CLIA and CAP Certified high complexity laboratory (Quest Diagnostics, San Juan Capistrano, CA) that ran a pharmacogenomic test panel for the purpose of identifying genotype and copy number variations. The PGx test encompasses genes and variants (Supplemental Table 1) that influence the pharmacokinetic or pharmacodynamic properties of medications. These genes were based on their documented clinical utility, their impact on medication use outcomes [1], and their inclusion in the Association for Molecular Pathology (AMP) PGx Working Group lists of recommended alleles for PGx testing. Results were converted into diplotypes based on standard nomenclatures [11–13] and the results were made available to the clinical decision support tool, GeneDose LIVE™ (Coriell Life Sciences, Philadelphia, PA). GeneDose LIVE™ interprets genotypes using known drug-gene interactions from guidelines, drug labels, and curated data from evidence-based literature.

### Medication action plan (MAP)

Coriell Life Science pharmacists utilized the comprehensive clinical decision support tool, GeneDose LIVE™, first to evaluate genetic and non-genetic sources of patient-specific risk associated with their current medication regimen, and then to model alternative choices that presented lower risks for inefficacy and safety concerns. The pharmacist created a summary medication action plan with proposed changes and notes containing clinical rationale that were subsequently communicated via secure email or fax to the patient’s preferred prescribing physician(s).

### Evaluation and outcomes

The analysis was divided into pre- and post-program timeframes based on the individual’s cohort. The intervention month was the medication action plan delivery date for the intervention cohort and the program launch date of March 2021 was used for the control cohort. For the intervention group, the pre-program period was defined as the number of months before the intervention, and the post-program period was defined as the number of months after the intervention. For the control group, the pre-program period was defined as the 13 months from February 2020 to February 2021; the post-program period was defined as the next 13 months. Participating members with fewer than six months post-program data were excluded. For both groups, individuals not continuously insured—defined by health plan coverage for the entire 26 months—were excluded.

Pre-program statistics for age, sex, geographic region, medical cost, Charlson Comorbidity Index, number of medications, and number of PGx-impacted medications were calculated based on de-identified medical and pharmacy insurance claims data from the health plan’s administrative claims database. Medical claim costs were aggregated by month to evaluate direct medical costs per member per month (PMPM) for each individual. As previously described [1], medical claims not impacted by PGx + CMM, with the potential to bias results in small sample sizes, were excluded (i.e., pregnancy, oncology, and non-medication-related trauma and injuries) from both the intervention and control groups. Pharmacy claims for high-cost medications determined to be outliers (≥3*σ*) were excluded. HRU metrics for inpatient, emergency, and outpatient service usage were also calculated from insurance claims. For the measurement of outcomes, the medical and pharmacy costs (PMPM) were averaged for the pre- and post-program period, and the HRU metrics were summed for the total count for each period.

### Statistical analysis

Propensity score matching by multivariable logistic regression using 4:1 nearest-neighbor matching with a caliper of 0.1 to model the probability of enrolling in the program among those in the participating group was used to create a suitable control group thus reducing both bias and confounding resulting from differences between the two cohorts. Covariates related to the outcomes of interest (i.e., age, sex, geographic region, number of medications, number of PGx-impacted medications, baseline medical cost (PMPM), and Charlson Comorbidity Index) were included in the model.

A doubly robust modeling approach was used to further reduce bias and confounding, which included the program participation indication and the covariates used in the propensity score model to estimate the outcomes of interest [14]. For the continuous cost per-month outcome metric, an adjusted linear regression model was fit to estimate the program effect on the overall medical and pharmacy average member cost per month, and the specific medical, pharmacy, inpatient, emergency, and outpatient post-program average member cost per month. For the HRU outcome metrics, adjusted negative binomial regression models were fit to estimate the program effect on the post-program HRU counts. An offset term was included to account for the differing number of post-program months for individuals. For all models, the program participation indicator coefficient is used to describe the estimated effect of participating in the program. When all other covariates are the same, the program participation indicator coefficient is the isolated program effect at the individual employee level. P values ≤ 0.05 were considered statistically significant, and 95% confidence intervals are included to analyze variation observed in the program effect estimates.

## Results

### Retrospective study: intervention and control assignments

De-identified administrative medical and pharmacy claims were used to evaluate outcomes using the available 26 months of data. Claims data were available for 3252 members, including 1084 employees who enrolled in the program and 2168 members who did not participate in the program between March 2021 and December 2021. In the ongoing program, an enrolled individual was defined as completing the program once the genetic sample kit was returned and a medication action plan was generated.

**Fig. 1:**
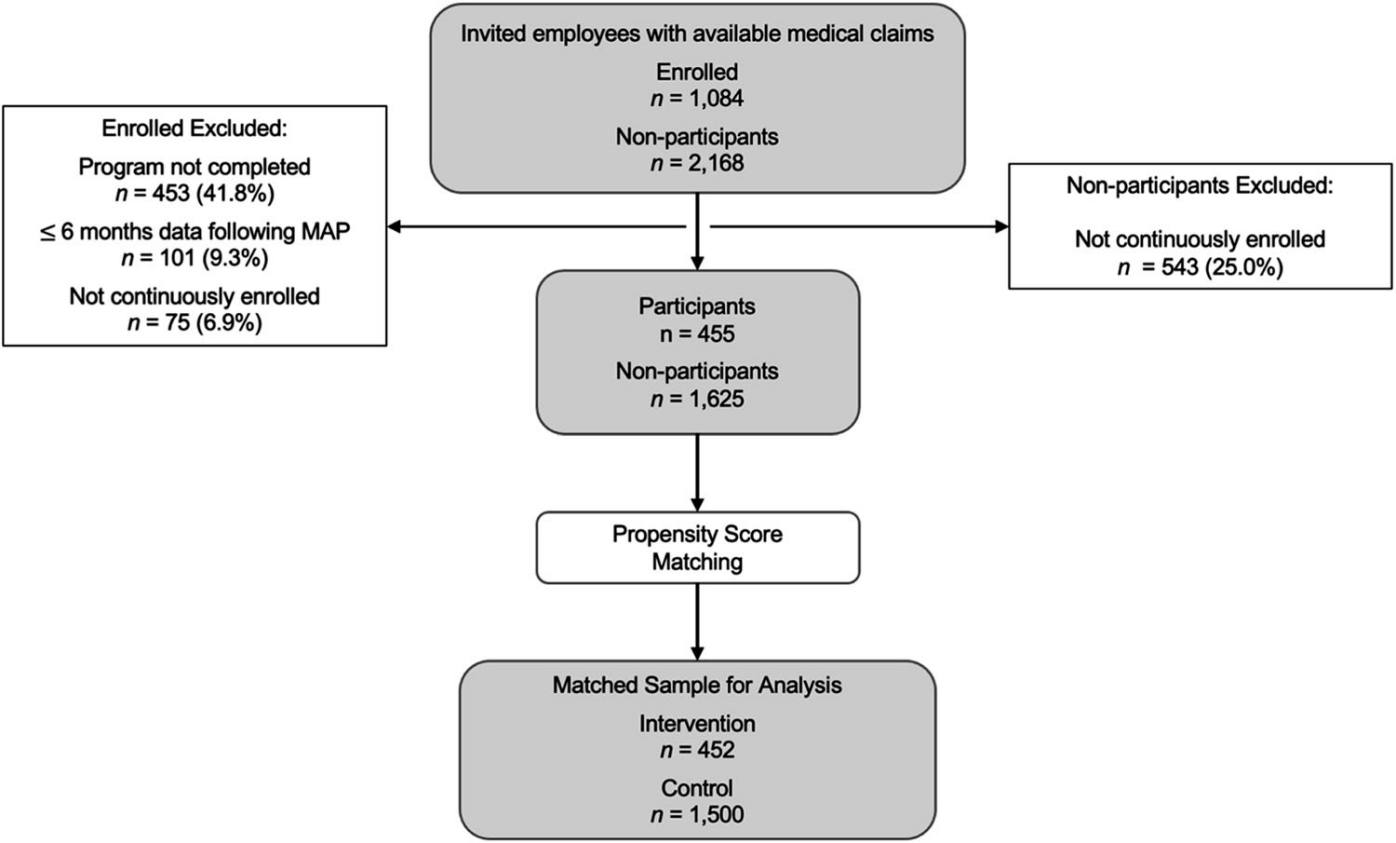
Flow diagram and cohort construction. Study population definitions and inclusion/exclusion criteria. Medical and pharmacy claims data were available for 3252 employees invited to the program. Following exclusion criteria (program completion, ≥6 months follow-up time, continuous enrollment), there were 455 participants and 1625 non-participants in the study population. Propensity score matching resulted in the final assignment of 452 to the intervention group and 1500 to the control group which were used for the clinical and economic analysis.

Claims were filtered to only the observation period. Following this, in the participant group, 1084 were enrolled, 631 completed the program, 530 had 6 or more months follow-up time, and 455 were continuously enrolled (Fig. 1). In the non-participant group, 1625 of the 2168 individuals were continuously enrolled. Before propensity score matching, the participant group used more PGx-impacted medications and had lower baseline cost than those who were invited but did not enroll in the program (Table 1). Both groups had similar numbers of overall prescriptions, 12.7 in the participant group and 12.3 in those who did not enroll (p = 0.28). The age, gender, number of medications, geographic region, and Charlson Comorbidity Index (CCI) scores were similar between the two groups. Propensity score matching resulted in 452 individuals assigned to the intervention group and 1500 individuals that were assigned to the control group for the evaluation. After matching, the intervention and control groups exhibited no differences in age, sex, geographic region, baseline medical cost, number of medications, number of PGx-impacted medications, and CCI score, as a result.

**Table 1:** Covariate comparison between groups before and after propensity score matching.

Variable	Participants (n = 455)	Non-participants (n = 1625)	P-value	Std mean diff	Intervention (n = 452)	Matched control group (n = 1500)	P-value	Std mean diff
*Demographics*								
Age	53	52.7	0.644	0.21	52.9	52.9	0.85	0.01
Sex								
Male	26.6%	24.7%	0.46	0.02	26.3%	25.8%	0.53	0.01
Female	73.4%	75.3%		0.04	73.7%	74.0%		0.01
Zip code region (0–9)								
0	10.6%	10.3%	0.56	0.01	10.4%	10.6%	0.89	0.01
1	7.5%	9.0%		0.06	7.5%	7.7%		0.01
2	13.6%	11.3%		0.07	13.7%	13.6%		0.00
3	17.4%	18.7%		0.03	17.5%	17.6%		0.00
4	10.3%	8.0%		0.08	10.2%	10.5%		0.01
5	1.0%	1.0%		0.01	1.0%	1.0%		0.01
6	10.6%	12.1%		0.05	10.6%	10.8%		0.01
7	15.6%	17.4%		0.05	15.7%	15.2%		0.01
8	5.3%	4.1%		0.05	5.1%	5.0%		0.01
9	8.6%	8.4%		0.01	8.6%	8.3%		0.01
*Risk Factors*								
Number of meds	12.7	12.3	0.28	0.06	12.6	12.6	0.43	0.00
Number of PGx meds	4.3	4	0.04	0.11	4.2	4.2	0.14	0.00
Baseline medical cost (PMPM)	1515.85	2117.47	0.03	0.18	1501.43	1558.62	0.87	0.02
CCI	1.5	1.6	0.68	0.02	1.5	1.6	0.63	0.02

*CCI* Charlson Comorbidity Index, *PMPM* per member per month, *PGx* Pharmacogenomics.

### Cost outcomes

The results of the adjusted linear regression models showed that participating in the program was associated with a decrease in total costs, including pharmacy and medical costs, of $128.31 PMPM (95% CI, −$646.44 to $389.81; p = 0.63) (Table 2). Similarly, program participation was associated with a decrease in medical-specific costs of $172.24 PMPM (95% CI, −$688.62 to $344.13; p = 0.51). Program participation was associated with an increase in pharmacy-specific costs of $26.30 PMPM (95% CI, $9.03–$43.56; p < 0.003). Program participation was associated with a decrease in costs specific to inpatient and emergency events of $1726.10 PMPM (95% CI, −$3383.71 to −$68.50; p = 0.04) and $33.36 PMPM (95% CI, −$70.28 to $3.56; p = 0.08), respectively. Program participation was associated with an increase in outpatient-specific costs of $114.51 PMPM (95% CI, −$296.56 to $525.57; p = 0.58).

**Table 2:** Program participation effect for healthcare costs ($PMPM) estimated by adjusted linear regression models.

Model	Program effect estimate ($)	P-Value	95% CI ($)
Total cost (medical + pharmacy)	−128.31	0.63	(−646.44, 389.81)
Medical cost	−172.24	0.51	(−688.62, 344.13)
Pharmacy cost	26.30	0.003	(9.03, 43.56)
IP only cost	−1726.10	0.04	(−3383.71, −68.50)
ED only cost	−33.36	0.08	(−70.28, 3.56)
OP only cost	114.51	0.58	(−296.56, 525.57)

*CI* Confidence interval, *ED* Emergency department, *IP* Inpatient, *OP* Outpatient, *PMPM* per member per month.

### Healthcare resource utilization outcomes

In the 13-month follow-up period, the results of the adjusted negative binomial regression models showed that program participation was associated with a decrease in inpatient (−39%, 95% CI, −63% to −1%; p = 0.05) and emergency (−39%, 95% CI, −56% to −16%; p = 0.002) visits and an increase in outpatient visits (21%, 95% CI, 13%–34%; p < 0.001) (Table 3).

**Table 3:** Program participation effect for healthcare resource utilization (% change) estimated by adjusted negative binomial regression models.

Model	Model coefficient	Program effect estimate	P-Value	95% CI
IP Events	−0.49	−39%	0.05	(−63%, −1%)
ED Events	−0.50	−39%	0.002	(−56%, −16%)
OP Events	0.21	21%	<0.001	(13%, 34%)

*CI* Confidence interval, *ED* Emergency department, *IP* Inpatient, *OP* Outpatient.

## Discussion

In a self-insured employee population, a medication safety program, consisting of PGx-enriched comprehensive medication management (PGx + CMM), resulted in favorable health outcomes in the year following the intervention. In the 13-month follow-up period, program participation was associated with significantly fewer inpatient and emergency department visits compared to the control group. In addition, the program showed potential economic benefits as measured by healthcare resource utilization (HRU) and costs in medical claims. These findings extend prior applications of real-world implementation of PGx + CMM [1] into a broader population and offer cost savings potential for self-insured employers opting to provide a similar medication safety program to employees. Additionally, results further support the attainment of a tipping point [2] for population-level, large-scale pharmacogenomic testing.

The findings show a positive shift in HRU away from acute, expensive services and towards less costly outpatient care settings. Reducing hospital inpatient and emergency department admissions represent a favorable impact on HRU since emergency department visits and inpatient hospital admissions drain healthcare resources and often indicate missed proactive and preventive care opportunities. Costs of inpatient care are rising and account for ~27% of privately paid healthcare expenditures [15]. Similarly, health spending attributable to emergency department visits is increasing in the U.S. and currently represents approximately 5% of total healthcare spending [16]. Outpatient visits reflect healthcare engagement in primary and preventive care services [17], higher utilization of evidence-based preventive health measures [18, 19], and an accepted strategy to prevent avoidable hospitalizations. In fact, observed increases in outpatient visits in the intervention group may be attributable to recommendations from program pharmacists for participants to follow-up with their healthcare providers. Findings suggest that recommendations were successfully communicated by the pharmacist to the patient’s prescribers, resulting in more optimized medication management leading to significantly attenuated HRU. Combined, the evidence supports that the PGx + CMM program favorably impacts HRU and offers a potential for cost savings at the population-level.

The total pharmacy and medical costs were estimated to decrease by $128.31 PMPM in the intervention group when compared to the control group. Medical-specific costs decreased by $172.24 PMPM for the intervention group. These savings estimates do not incorporate the cost of the program. While reductions in total medical costs were not statistically significant due to high variability in costs, observed savings track with the shift away from inpatient and emergency department services and are consistent with previous studies [1, 20]. Additionally, pharmacy costs increased by $26.30 PMPM for the intervention group—not an unexpected result as healthcare providers may habitually prescribe less expensive medication before moving on to more costly alternatives. Prior to the intervention, the participants had lower total medical costs likely due to lower utilization of costly inpatient and emergency services compared to the control. However, even after matching for baseline differences in total medical costs between the groups, the PGx + CMM program was associated with a decrease in total medical costs for the intervention group.

These results build on the compelling evidence of the clinical and economic value of introducing PGx + CMM as a standard of care by expanding to a younger, employed population. While participants were invited into the program based on risk and utilization of medications with PGx implications, PGx interventions may have wider population impact as almost 65% of US adults may be exposed to at least one medication with an established pharmacogenomic association within a 5-year window [21]. Further, it is estimated that 99% of individuals harbor a DNA variant known to impact medication safety and efficacy [22]. This paper suggests that provisioning a PGx + CMM program across similar populations would yield positive clinical and economic impacts. Together, these outcomes provide evidence of a successful PGx + CMM implementation model.

At present, coverage for pharmacogenomics testing across most commercial plans is limited and reimbursement at the individual level continues to lag. This may be due—at least in part— to the uncertain regulatory environment regarding pharmacogenomic testing in the US. However, distinct from complicated insurance-based provisions is the opportunity for employers to implement a PGx + CMM program, thus no longer relying on third-party payers to reimburse for PGx testing and medication management services. Employers can thus remove third-party reimbursement constraints while saving costs and improving the health of their employees. Furthermore, PGx + CMM implementations are being initiated by single-payer healthcare systems and countries [23] lending additional credence to population-level PGx + CMM programs. These all suggest that, given no mechanism to fund this population-level activity within the established process of medical necessity decision-making at the patient level, self-insured employers, with more control over healthcare spending, might be interested in offering this program for employees. Given additional opportunities to increase PGx + CMM program accessibility through an employer channel, it would be beneficial to further explore this implementation method.

Despite the positive results of the current report, findings should be interpreted in context and with an understanding of limitations. First, this paper reports findings of a real-world implementation as opposed to a randomized controlled trial. Although randomized controlled trials may provide the highest level of evidence, in clinical evaluations of pharmacogenomics, ethical considerations arise regarding the assignment of individuals with pharmacogenetic risk to a control group [24]. Specifically, such considerations may deem that it is not ethical to deny a person with known risk access to an intervention known to be beneficial. It is also noted that real-life clinical evaluations carry the benefit of higher external validity with more transferable evidence to everyday clinical practice, despite any perceived lower level of evidence [24]. Future studies with larger sample sizes and extended program durations could provide more evidence to support the findings of this paper. Specifically, these studies could explore how changes in healthcare resource utilization relate to disease severity, care needs, and clinical outcomes. Although our analysis suggests a strong link between the PGx + CMM intervention and the observed outcomes, we cannot conclusively establish a causal connection between specific medication changes and healthcare resource utilization in this study. Yet, we may reasonably assume that pharmacist-recommended changes were implemented given our previous report showing that 86% of employees who completed the program received actionable recommendations, averaging 5 recommendations per person [10]. Never-the-less, a deeper understanding of the relationship between the intervention and outcomes could enhance future research. Additionally, this implementation was a voluntary employer-sponsored program, so selection bias may have contributed to the observed findings. However, this bias was addressed through statistical methodology and propensity score matching. Further research regarding why some employees, and not others, voluntarily participated in the intervention could enable more targeted engagement in future implementations. Moreover, several additional factors that have been associated with HRU, such as demographic, socioeconomic, health services–related, health status–related, and health insurance coverage, were not evaluated in the present study [25]. In addition, individuals were invited to the program based on risk stratification and evidence of using ≥1 prescription medication. It may be that implementation in populations with lower medication utilization at baseline yields different results. However, potential differences can be mitigated by using a risk stratification process to identify individuals within the population most likely to benefit from the program. Finally, although the value of PGx-guided treatment has not been easily compared across different genetic assays and implementation strategies, our study aligns with a growing body of research demonstrating that pharmacogenetic testing, when integrated with clinical decision support, can lead to improved healthcare utilization and potential cost savings in the management of polypharmacy [26].

## Conclusions

Pharmacogenomics-enriched comprehensive medication management can favorably impact healthcare utilization in a self-insured employer population by reducing emergency department and inpatient visits and can offer the potential for cost savings. Self-insured employers may consider implementing pharmacogenomics-enriched comprehensive medication management to improve the healthcare of their employees [27].

## Supplementary information

Supplementary Table 1: Genes Evaluated for the Pharmacogenomics-enriched Comprehensive Medication Management Program

## Data Availability

The data are not publicly available due to their containing information that could compromise the privacy of program participants.
