# Supplementary material for: Clinical and economic outcomes of a pharmacogenomics-enriched comprehensive medication management program in a self-insured employee population
Source: Pharmacogenomics J. 2024 Oct 2;24(5):30. doi: 10.1038/s41397-024-00350-1 (PMC11446811; doi:10.1038/s41397-024-00350-1)
Supplement: Supplementary file 1 — Supplementary Table 1: Genes Evaluated for the Pharmacogenomics-enriched Comprehensive Medication Management Program [file 41397_2024_350_MOESM1_ESM.docx]

Fragala et al., *Clinical and Economic Outcomes of a Pharmacogenomics-enriched Comprehensive Medication Management Program in a Self-insured Employee Population*

**Supplemental Table 1: Genes Evaluated for the Pharmacogenomics-enriched Comprehensive Medication Management Program**

| **Assayed Gene** | **Evaluated Variants (SNP/Alleles)** |
| --- | --- |
| ABCG2 | rs2231142 |
| ADRA2A | rs1800544 (c.-1252G>C) |
| ANKK1 | rs1800497 (c.2137G>A) |
| ATM (C11orf65) | rs11212617 |
| COMT | rs4680 (c.472G>A; Val158Met) |
| CYP2B6 | *5, *6, *7, *16, *22, *34 |
| CYP2C9 | *2, *3, *4, *5, *6, *8, *11, *27 |
| CYP2C19 | *2, *3, *4A, *4B, *5, *6, *7, *8, *9, *10, *17 |
| CYP2D6 | *2, *4, *5, *6, *7, *8, *9, *10, *11, *12, *14, *15, *17, *29, *35, *36, *41, *64, *69, *82, *91, *109, *114, CNVs (duplications) |
| CYP3A4 | *1B, *2, *3, *12, *17, *22 |
| CYP3A5 | *2, *3B, *3C, *6, *7, *8, *9 |
| CYP4F2 | *3 |
| DPYD | *2A, *5, *7, *8, *9B, *10, *13, 1129-5923C>G, HapB3 |
| F5 | rs6025 (Factor V Leiden) |
| HLA-B | *15:02, *57:01 |
| IFNL3 | rs12979860 |
| NUDT15 | *3 |
| OPRM1 | rs1799971 (c.118A>G) |
| SLCO1B1 | *5 |
| TPMT | *2, *3A, *3B, *3C |
| VKORC1 | *2 (-1639G>A) |
